# Short-term and Long-term Mortality Following Hospitalized and Ambulatory Lower Respiratory Tract Illnesses Among US Adults

**DOI:** 10.1093/ofid/ofaf186

**Published:** 2025-03-26

**Authors:** Ahuva Averin, Reiko Sato, Elizabeth Begier, Kari Yacisin, Linnea Houde, Alexander Lonshteyn, Derek Weycker

**Affiliations:** Avalere Health, Boston, Massachusetts, USA; Pfizer Inc., Collegeville, Pennsylvania, USA; Pfizer Inc., Collegeville, Pennsylvania, USA; Pfizer Inc., Collegeville, Pennsylvania, USA; Avalere Health, Boston, Massachusetts, USA; Avalere Health, Boston, Massachusetts, USA; Avalere Health, Boston, Massachusetts, USA

**Keywords:** mortality, pneumonia, respiratory syncytial viruses, respiratory tract infections, administrative claims, healthcare

## Abstract

**Background:**

Lower respiratory tract illness (LRTI) is a significant cause of morbidity among adults, particularly older adults and adults with underlying medical conditions. Evidence on short- and long-term risks of mortality among adults requiring hospitalization or ambulatory care for LRTI, overall and within subgroups, is currently lacking.

**Methods:**

A retrospective observational matched-cohort design and Optum's de-identified Clinformatics Data Mart Database (2012–2019) were used. The study population included adults who were hospitalized or received ambulatory care for LRTI and matched (1:1) comparison patients. All-cause mortality was ascertained during the 30-, 60-, 90-, 180-, and 360-day periods following the beginning of the LRTI episode. Risks of mortality were estimated for all LRTI patients and comparison patients as well as within age/comorbidity-specific subgroups.

**Results:**

Among LRTI-hospitalized patients (n = 60.2K), 30-day mortality risk was 5.8% and 360-day risk was 18.3%, 7.5 and 2.6 times higher than corresponding values for comparison patients. Among LRTI-ambulatory patients (n = 2.4M), 30-day mortality risk was 1.2% and 360-day risk was 3.6%, 6.5 and 2.1 times higher than comparison patients. Among both LRTI-hospitalized and LRTI-ambulatory patients, mortality risk increased with increasing age and was higher for adults with chronic or immunocompromising conditions (vs without medical conditions).

**Conclusions:**

Short- and long-term mortality were higher among patients who were hospitalized or received ambulatory care for LRTI vs matched comparison patients, and risks increased markedly with increasing age and worsening comorbidity profile. Strategies for preventing LRTI, especially among persons at elevated risk, may reduce premature deaths and yield important public health benefits.

## Teaser

Study findings indicate that hospitalized and ambulatory patients treated for lower respiratory tract illness had elevated rates of mortality (versus matched comparison patients) in both short- and long-term analyses; mortality rates were highest in older adults and those with comorbidities.

Acute infections of the lower respiratory tract include those of the lung (pneumonia) and airways (ie, acute bronchitis and acute bronchiolitis) and are often caused by viral pathogens (eg, respiratory syncytial virus [RSV], influenza) and/or bacterial pathogens (eg, *Pneumococcus*) [1, 2]. Lower respiratory tract illness (LRTI) is a significant cause of morbidity and mortality in the United States, particularly among older adults and adults of all ages with underlying medical conditions [1, 3–5]. Risks of mortality among patients hospitalized with LRTI—irrespective of clinical manifestation and causative pathogen—are known to be high during the acute phase of illness as well as during the 1-year period following diagnosis and to increase with increasing age.

Two recent studies of patients with all-cause community-acquired pneumonia reported in-hospital mortality between 3.5% and 6.5%, 30-day mortality between 8.2% and 13.0%, and 1-year mortality between 17.7% and 30.6% [6, 7]. Moreover, Averin et al. found that mortality risk increased monotonically with age and severity of comorbidity profile [7]. Similar findings have been reported in other published studies focusing on adults hospitalized for or with influenza, RSV, and other infectious diseases [8–10]. However, few studies have evaluated short- and long-term mortality among younger and older adults who were hospitalized for LRTI within subgroups defined on age and comorbidity profile [6–10]. Moreover, to the best of our knowledge, none have evaluated short/long-term mortality among patients with LRTI stratified by care setting (ie, hospitalized vs ambulatory), age, and comorbidity profile relative to a matched comparison group. We therefore undertook a new study using a large US health care claims database to address these evidence gaps.

## METHODS

### Study Design and Data Source

This study used a retrospective observational matched-cohort design and data from Optum's de-identified Clinformatics Data Mart Database (CDM), which was linked to date of death from the Social Security Administration (SSA) Death Master File. Data for this study spanned from January 1, 2012, through December 31, 2019; the study period end date was selected to avoid potential confounding of study results from use of data during the COVID-19 pandemic. The Clinformatics Datamart comprises medical (ie, facility and professional service) and outpatient pharmacy claims from a large US private health plan covering >12.5 million geographically diverse members annually, including enrollees, their spouses, and their dependents. Adults aged ≥65 years who have elected to enroll in a Medicare Advantage Plan—and thus receive their health care coverage through a private health plan—also are included in the data source population.

Data available from each medical claim include dates and places of service, diagnoses, procedures performed/services rendered, and quantity of services (professional service claims only). Data available for each outpatient pharmacy claim include the drug dispensed, dispensing date, quantity dispensed, and number of days supplied. Medical and pharmacy claims also include standardized costs, which represent estimates of allowed amounts for facility or professional service charges, and selected demographic and eligibility information is available. The study database was de-identified before its release to study investigators, as set forth in the corresponding Data Use Agreement. Use of the study database for health services research was fully compliant with the HIPAA Privacy Rule and federal guidance on Public Welfare and the Protection of Human Subjects (45 CFR 46 §46.101). Institutional review board (IRB) approval and informed consent were not required.

### Study Population

The study population included adults aged ≥18 years with a new episode of LRTI between January 1, 2013, and December 31, 2018 (“LRTI patients”), as well as matched “comparison patients.” All adults designated as “LRTI patients” satisfied the following criteria: evidence of a new episode of LRTI requiring acute care hospitalization or care in an ambulatory setting (ie, emergency department, physician office/hospital outpatient [PO/HO]); continuous and comprehensive health care coverage during the 12-month (“history”) period before the beginning of the LRTI episode (“index date”); and no evidence of LRTI during the 60-day period before the “index” LRTI episode.

All qualifying LRTI encounters occurring within 60 days of each other were considered part of the same episode, and qualifying encounters separated by >60 days were considered separate episodes. Episodes including a hospitalization for LRTI (irrespective of ambulatory LRTI encounters during the episode) that occurred within 60 days of the index date were considered a hospitalized episode, and the index date was redefined as the date of hospital admission. Hospitalized LRTI was identified based on an acute care inpatient admission with a corresponding diagnosis code (Supplementary Appendix A) in the principal position on an inpatient facility claim. Ambulatory LRTI was identified based on an outpatient encounter with a corresponding diagnosis code in any position. Hospitalized LRTI patients were excluded from the study population if they were transferred from another hospital.

Each LRTI patient was matched on a monthly basis (starting January 2013) to 1 “comparison patient” from the source population who, during the 1-year period before the new LRTI episode (ie, for the LRTI patient), had continuous and comprehensive health benefits and no evidence of LRTI (including the match month). Each comparison patient was assigned the same index date as the LRTI patient to whom he/she was matched. Once matched, both the LRTI patient and the comparison patient were included in the study population and removed from the source population. The same process was repeated for each subsequent calendar month—using the pool of patients remaining in the source population after matching in prior months—ending with December 2018.

Matching was implemented for each LRTI patient by first identifying all “candidate” comparison patients based on the aforementioned criteria as well as age, sex, comorbidity profile, and selected markers for health care burden (see “Patient Characteristics”). Among all such candidates, one was randomly selected for matching and included in the study population.

### Study Variables

#### Study Outcome

Death (all-cause) was ascertained during the 30-day, 60-day, 90-day, 180-day, and 360-day periods, respectively, from the index date (ie, beginning of the LRTI episode [inclusive]), as defined above.

#### Baseline Characteristics

Baseline characteristics of the study population included age, sex, comorbidity profile, and markers of health care burden. Comorbidity profile was defined as “immunocompetent without a chronic medical condition” (CMC−), “immunocompetent with a chronic medical condition” (CMC+), and “immunocompromised” (IC) and was ascertained during the 12-month history period based on the presence (vs absence) of medical conditions listed in the US Advisory Committee on Immunization Practices (ACIP) recommendations for influenza vaccination (Supplementary Appendix B) [11]. Markers of health care burden were ascertained during the 12-month history period and included categorical variables for number of all-cause ambulatory encounters, number of all-cause hospitalizations, and total all-cause health care costs.

### Statistical Methods

Baseline characteristics of LRTI patients and comparison patients were summarized using descriptive statistics (eg, means/SDs, percentages). Mortality risks for LRTI patients and comparison patients were summarized using incidence proportions and corresponding 95% CIs, which were calculated using the Wilson score interval. Analyses were conducted for LRTI patients and matched comparison patients by care setting, on an overall basis, and within subgroups defined therein on age (18–49, 50–64, 65–74, 75–84, 85–99 years) and comorbidity profile (CMC−, CMC+, IC). LRTI-associated mortality was calculated based on the difference in estimated mortality risks between LRTI patients and matched comparison patients.

## RESULTS

### Hospitalized LRTI

#### Patient Characteristics

Among the 170 535 adults aged ≥18 years who were hospitalized for LRTI during 2013–2018, 80 575 met the minimum enrollment criterion (≥12 months pre-LRTI), and among these patients, 60 208 met all remaining selection criteria and were matched to comparison patients. The mean (SD) age of hospitalized LRTI patients was 71.0 (14.4) years, and nearly 75% of patients were designated as CMC+ (58.5%) or IC (15.8%); 89.9% of hospitalized LRTI patients had evidence of pneumonia (Table 1). Baseline characteristics of hospitalized LRTI patients (as well as ambulatory LRTI patients) and matched comparison patients by age and comorbidity profile are set forth in Supplementary Tables 1 and 2.

**Table 1.** Baseline Characteristics of Hospitalized LRTI Patients and Ambulatory LRTI Patients (and Matched Comparison Patients^a^)

	LRTI-Hospitalized Patients (n = 60 208)	LRTI-Ambulatory Patients (n = 2 429 188)
Demographic profile		
Age, mean (SD), y	71.0 (14.4)	53.8 (18.3)
Age groups, %		
18–49 y	8.7	41.0
50–64 y	18.5	26.3
65–74 y	25.7	18.1
75–84 y	30.1	11.3
≥85 y	17.1	3.3
Sex, %		
Male	45.5	43.8
Female	54.5	56.2
Comorbidity profile, %		
CMC−	25.7	64.4
CMC+	58.5	29.4
Cardiopulmonary	0.9	0.2
Cardiovascular	39.3	13.5
Hematologic	6.4	1.6
Hepatic	0.8	0.5
Metabolic	31.5	15.5
Neurologic	19.8	5.9
Pulmonary	24.9	10.2
Renal	15.3	4.6
Obesity (BMI >40 kg/m^2^)	3.4	2.2
IC	15.8	6.2
Health care profile		
No. of all-cause ambulatory encounters, %		
0	1.9	5.2
1–9	18.8	40.8
10–19	22.3	25.1
≥20	57.0	28.9
No. of all-cause hospitalizations, %		
0	74.0	90.5
1	13.8	6.5
≥2	12.2	3.0
Health care costs, %		
<$1000	11.0	30.3
$1000–$4999	25.2	34.3
$5000–$9999	14.8	12.8
$10 000–$19 999	14.4	9.6
$20 000–$49 999	16.7	7.8
≥$50 000	17.9	5.2

Abbreviations: BMI, body mass index; CMC, chronic medical conditions; IC, immunocompromised; LRTI, lower respiratory tract infection.

^a^LRTI and comparison patients were matched 1:1 on baseline characteristics; thus means and percentages are identical between subgroups.

#### Risk of Mortality

**Figure 1.**
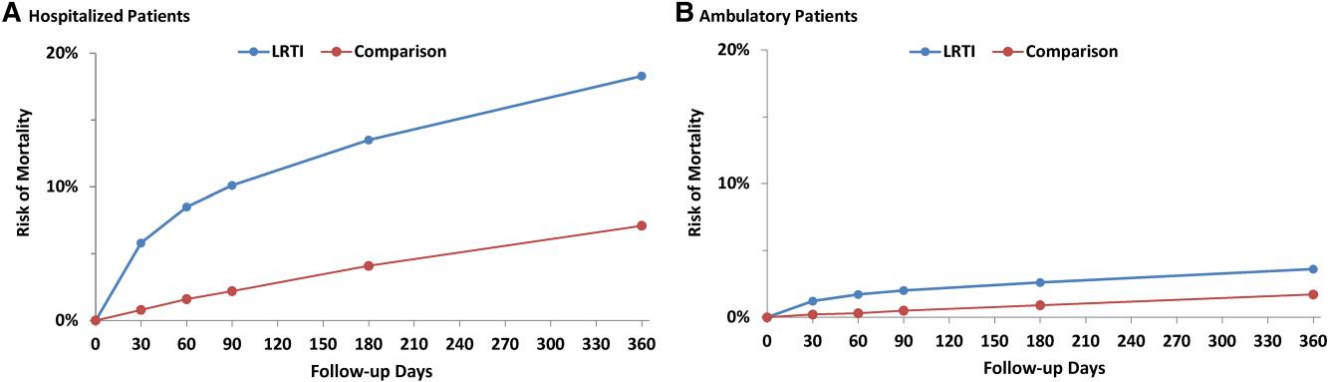
Risk of mortality (all-cause) among (*A*) hospitalized and (*B*) ambulatory LRTI patients and matched comparison patients. Abbreviation: LRTI, lower respiratory tract illness.

Risk of mortality among hospitalized LRTI patients was 5.8% (5.7%–6.0%) at 30 days, 13.5% (13.3%–13.8%) at 180 days, and 18.3% (18.0%–18.6%) at 360 days, 7.5, 3.3, and 2.6 times higher than the corresponding values for matched comparison patients (Table 2, Figure 1). LRTI-associated mortality risks were thus 5.1% at 30 days, 9.5% at 180 days, and 11.2% at 360 days. Risk of mortality among hospitalized LRTI patients increased with increasing age from 18–49 years to ≥85 years: from 1.1% (0.8%–1.4%) to 11.9% (11.2%–12.5%) at 30 days and from 3.1% (2.7%–3.6%) to 35.0% (34.1%–36.0%) at 360 days. Age-specific relative risks (vs comparison patients) ranged from 4.8 (≥85 years) to 56.0 (18–49 years) at 30 days and from 1.6 (≥85 years) to 6.0 (18–49 years) at 360 days; LRTI-associated risks ranged from 1.1% (18–49 years) to 9.4% (≥85 years) at 30 days and from 2.6% (18–49 years) to 14.1% (75–84 years) at 360 days.

**Table 2.** Risk of Mortality (All-Cause) Among Hospitalized LRTI Patients as Well as Matched Comparison Patients

		Risk of Mortality (95% CI), %														
	No. of Matched Pairs	30 Days			60 Days			90 Days			180 Days			360 Days		
		LRTI Pts	Comp. Pts	RR	LRTI Pts	Comp. Pts	RR	LRTI Pts	Comp. Pts	RR	LRTI Pts	Comp. Pts	RR	LRTI Pts	Comp. Pts	RR
Overall	60 208	5.8 (5.7–6.0)	0.8 (0.7–0.9)	7.5 (6.8–8.2)	8.5 (8.3–8.7)	1.6 (1.5–1.7)	5.4 (5.0–5.8)	10.1 (9.9–10.4)	2.2 (2.1–2.3)	4.6 (4.3–4.8)	13.5 (13.3–13.8)	4.1 (3.9–4.2)	3.3 (3.2–3.5)	18.3 (18.0–18.6)	7.1 (6.9–7.3)	2.6 (2.5–2.7)
age																
18–49 y	5211	1.1 (0.8–1.4)	0.0 (0.0–0.1)	56.0 (7.8–404.4)	1.4 (1.1–1.8)	0.1 (0.0–0.2)	14.6 (5.9–36.1)	1.6 (1.3–2.0)	0.1 (0.1–0.3)	11.9 (5.5–25.6)	2.3 (1.9–2.7)	0.3 (0.2–0.5)	8.0 (4.7–13.7)	3.1 (2.7–3.6)	0.5 (0.4–0.8)	6.0 (4.0–8.9)
50–64 y	11 147	2.5 (2.2–2.8)	0.3 (0.2–0.4)	8.8 (6.1–12.6)	3.9 (3.6–4.3)	0.6 (0.5–0.8)	6.4 (5.0–8.3)	4.8 (4.4–5.2)	0.8 (0.6–1.0)	6.2 (4.9–7.7)	6.6 (6.2–7.1)	1.4 (1.2–1.6)	4.9 (4.1–5.8)	9.1 (8.6–9.6)	2.3 (2.1–2.6)	3.9 (3.4–4.5)
65–74 y	15 478	4.9 (4.5–5.2)	0.4 (0.3–0.5)	12.9 (9.9–16.9)	7.1 (6.7–7.5)	0.8 (0.7–1.0)	8.8 (7.4–10.6)	8.7 (8.2–9.1)	1.2 (1.0–1.4)	7.4 (6.4–8.6)	11.8 (11.3–12.3)	2.0 (1.8–2.2)	5.9 (5.2–6.6)	16.0 (15.5–16.6)	3.7 (3.4–4.0)	4.4 (4.0–4.8)
75–84 y	18 100	6.7 (6.3–7.1)	0.7 (0.6–0.8)	9.5 (8.0–11.4)	9.7 (9.3–10.2)	1.4 (1.3–1.6)	6.8 (5.9–7.7)	11.4 (10.9–11.9)	2.0 (1.8–2.2)	5.8 (5.2–6.5)	15.2 (14.7–15.7)	3.6 (3.3–3.9)	4.2 (3.9–4.6)	20.8 (20.2–21.4)	6.7 (6.3–7.0)	3.1 (2.9–3.3)
≥85 y	10 272	11.9 (11.2–12.5)	2.5 (2.2–2.8)	4.8 (4.2–5.5)	17.0 (16.3–17.8)	4.8 (4.4–5.2)	3.6 (3.2–3.9)	20.3 (19.6–21.1)	6.9 (6.4–7.4)	2.9 (2.7–3.2)	26.5 (25.7–27.4)	12.9 (12.2–13.5)	2.1 (1.9–2.2)	35.0 (34.1–36.0)	21.3 (20.5–22.1)	1.6 (1.6–1.7)
Comorbidity profile																
CMC−	15 495	2.9 (2.7–3.2)	0.2 (0.1–0.2)	17.4 (11.7–25.9)	4.1 (3.8–4.5)	0.4 (0.3–0.5)	11.3 (8.6–14.8)	4.9 (4.5–5.2)	0.6 (0.5–0.8)	7.7 (6.2–9.5)	6.3 (6.0–6.7)	1.2 (1.0–1.4)	5.4 (4.6–6.3)	8.3 (7.9–8.7)	2.1 (1.9–2.3)	3.9 (3.5–4.4)
CMC+	35 207	5.9 (5.7–6.2)	0.9 (0.8–1.0)	6.9 (6.1–7.8)	8.5 (8.3–8.8)	1.7 (1.6–1.8)	5.0 (4.6–5.5)	10.2 (9.9–10.5)	2.4 (2.2–2.6)	4.3 (4.0–4.6)	13.8 (13.4–14.1)	4.5 (4.3–4.8)	3.0 (2.9–3.2)	19.1 (18.7–19.5)	8.1 (7.8–8.4)	2.4 (2.3–2.5)
IC	9506	10.4 (9.8–11.0)	1.5 (1.3–1.8)	6.8 (5.7–8.1)	15.5 (14.8–16.2)	3.1 (2.8–3.5)	5.0 (4.4–5.6)	18.4 (17.7–19.2)	4.2 (3.8–4.6)	4.4 (4.0–4.9)	24.4 (23.6–25.3)	7.0 (6.5–7.5)	3.5 (3.2–3.8)	31.6 (30.7–32.5)	11.3 (10.7–12.0)	2.8 (2.6–3.0)
Age and risk																
18–49 y																
CMC−	3261	0.6 (0.4–1.0)	0.0 (0.0–0.0)	∞	0.8 (0.5–1.1)	0.0 (0.0–0.0)	∞	0.8 (0.5–1.2)	0.0 (0.0–0.0)	∞	1.0 (0.7–1.5)	0.0 (0.0–0.0)	∞	1.4 (1.1–1.9)	0.0 (0.0–0.0)	∞
CMC+	1457	0.9 (0.5–1.5)	0.0 (0.0–0.0)	∞	1.2 (0.7–1.9)	0.0 (0.0–0.0)	∞	1.4 (0.9–2.1)	0.1 (0.0–0.5)	20.0 (2.7–148.8)	2.4 (1.7–3.3)	0.3 (0.1–0.8)	7.0 (2.8–17.8)	3.4 (2.6–4.4)	1.0 (0.6–1.6)	3.5 (1.9–6.3)
IC	493	4.5 (3.0–6.7)	0.2 (0.0–1.4)	22.0 (3.0–162.6)	6.3 (4.5–8.8)	1.0 (0.4–2.4)	6.2 (2.4–15.8)	7.5 (5.5–10.2)	1.2 (0.5–2.7)	6.2 (2.6–14.5)	10.3 (8.0–13.4)	2.0 (1.1–3.7)	5.1 (2.6–9.9)	13.2 (10.5–16.5)	2.6 (1.5–4.5)	5.0 (2.8–9.0)
50–64 y																
CMC−	3701	1.1 (0.8–1.5)	0.1 (0.0–0.3)	13.3 (4.1–43.1)	1.6 (1.3–2.1)	0.1 (0.1–0.3)	12.2 (4.9–30.3)	2.0 (1.6–2.5)	0.1 (0.1–0.3)	15.0 (6.1–37.0)	2.6 (2.2–3.2)	0.2 (0.1–0.4)	12.2 (6.0–25.1)	3.2 (2.6–3.8)	0.3 (0.2–0.6)	9.7 (5.4–17.6)
CMC+	5496	1.9 (1.6–2.3)	0.2 (0.1–0.4)	8.9 (4.9–16.2)	3.0 (2.6–3.5)	0.5 (0.4–0.8)	5.5 (3.7–8.1)	3.6 (3.1–4.1)	0.7 (0.5–1.0)	5.1 (3.6–7.1)	5.2 (4.6–5.8)	1.2 (1.0–1.5)	4.2 (3.3–5.5)	7.8 (7.1–8.5)	2.3 (1.9–2.7)	3.4 (2.8–4.1)
IC	1950	6.9 (5.8–8.1)	0.9 (0.5–1.4)	7.9 (4.8–13.0)	10.9 (9.6–12.3)	1.7 (1.2–2.4)	6.4 (4.5–9.2)	13.2 (11.8–14.8)	2.2 (1.6–2.9)	6.1 (4.4–8.4)	18.3 (16.7–20.1)	3.9 (3.1–4.9)	4.7 (3.7–6.0)	24.0 (22.2–26.0)	6.1 (5.1–7.3)	3.9 (3.2–4.8)
65–74 y																
CMC−	3286	2.5 (2.0–3.1)	0.1 (0.0–0.2)	40.5 (10.0–164.6)	3.3 (2.7–4.0)	0.2 (0.1–0.4)	21.6 (8.8–52.9)	3.7 (3.1–4.5)	0.2 (0.1–0.5)	15.4 (7.5–31.4)	5.0 (4.3–5.8)	0.4 (0.2–0.6)	13.7 (7.6–24.5)	6.8 (6.0–7.7)	0.7 (0.5–1.1)	9.7 (6.3–14.9)
CMC+	9335	3.9 (3.5–4.3)	0.3 (0.2–0.4)	14.4 (9.6–21.6)	5.8 (5.3–6.3)	0.5 (0.4–0.7)	10.6 (7.9–14.1)	7.1 (6.6–7.6)	0.8 (0.6–1.0)	9.0 (7.1–11.4)	9.9 (9.3–10.5)	1.5 (1.3–1.8)	6.4 (5.4–7.6)	13.9 (13.2–14.6)	2.9 (2.6–3.3)	4.8 (4.2–5.4)
IC	2857	10.8 (9.7–12.0)	1.1 (0.8–1.5)	10.0 (6.9–14.4)	16.0 (14.7–17.4)	2.4 (1.9–3.0)	6.6 (5.2–8.5)	19.5 (18.1–21.0)	3.5 (2.9–4.2)	5.6 (4.6–6.9)	25.9 (24.3–27.5)	5.4 (4.7–6.3)	4.8 (4.0–5.6)	33.7 (32.0–35.4)	9.5 (8.4–10.6)	3.6 (3.1–4.0)
75–84 y																
CMC−	3368	3.6 (3.0–4.3)	0.1 (0.0–0.3)	40.3 (12.8–126.7)	5.3 (4.6–6.2)	0.1 (0.0–0.3)	45.0 (16.7–121.1)	6.5 (5.7–7.4)	0.1 (0.1–0.4)	43.8 (18.1–106.1)	8.5 (7.6–9.5)	0.5 (0.3–0.8)	17.9 (10.8–29.5)	11.1 (10.1–12.2)	1.5 (1.1–1.9)	7.6 (5.7–10.2)
CMC+	11 641	6.2 (5.8–6.7)	0.6 (0.5–0.8)	10.3 (8.1–13.2)	8.9 (8.4–9.4)	1.3 (1.1–1.5)	6.7 (5.7–8.0)	10.4 (9.9–11.0)	1.8 (1.6–2.1)	5.8 (5.0–6.7)	14.2 (13.6–14.8)	3.4 (3.1–3.8)	4.2 (3.7–4.6)	20.1 (19.4–20.8)	6.7 (6.3–7.2)	3.0 (2.8–3.2)
IC	3091	11.9 (10.8–13.1)	1.7 (1.3–2.3)	6.8 (5.1–9.0)	17.7 (16.4–19.1)	3.3 (2.8–4.0)	5.3 (4.3–6.5)	20.4 (19.0–21.9)	4.6 (3.9–5.4)	4.5 (3.8–5.3)	26.2 (24.7–27.8)	7.5 (6.7–8.5)	3.5 (3.0–4.0)	33.9 (32.2–35.6)	12.1 (11.0–13.3)	2.8 (2.5–3.1)
≥85 y																
CMC−	1879	10.1 (8.8–11.6)	1.0 (0.6–1.5)	10.6 (6.5–17.0)	14.3 (12.8–16.0)	2.3 (1.7–3.1)	6.3 (4.6–8.6)	16.4 (14.9–18.2)	4.3 (3.4–5.3)	3.9 (3.0–4.9)	21.2 (19.5–23.2)	7.8 (6.6–9.1)	2.7 (2.3–3.3)	27.8 (25.8–29.9)	12.9 (11.5–14.5)	2.1 (1.9–2.5)
CMC+	7278	12.0 (11.3–12.8)	2.7 (2.3–3.1)	4.5 (3.9–5.2)	17.3 (16.4–18.1)	5.0 (4.5–5.5)	3.5 (3.1–3.9)	20.7 (19.8–21.7)	7.1 (6.6–7.8)	2.9 (2.6–3.2)	26.9 (25.9–28.0)	13.5 (12.8–14.3)	2.0 (1.9–2.1)	36.0 (34.9–37.1)	22.6 (21.7–23.6)	1.6 (1.5–1.7)
IC	1115	13.8 (11.9–16.0)	3.8 (2.8–5.1)	3.7 (2.6–5.1)	20.0 (17.8–22.5)	7.6 (6.2–9.4)	2.6 (2.1–3.3)	24.3 (21.9–27.0)	9.8 (8.2–11.7)	2.5 (2.0–3.1)	33.0 (30.4–35.9)	17.0 (14.9–19.3)	1.9 (1.7–2.3)	41.3 (38.5–44.2)	27.0 (24.5–29.7)	1.5 (1.4–1.7)

Abbreviations: CI, confidence interval; CMC, chronic medical conditions; IC, immunocompromised; LRTI, lower respiratory tract infection; Pts, patients; RR, relative risk.

Mortality risks among hospitalized LRTI patients also increased with more complex comorbidity profiles: 2.9% (2.7%–3.2%) for CMC−, 5.9% (5.7%–6.2%) for CMC+, 10.4% (9.8%–11.0%) for IC at 30 days; 8.3% (7.9%–8.7%) for CMC−, 19.1% (18.7%–19.5%) for CMC+, 31.6% (30.7%–32.5%) for IC at 360 days. At 30 days, relative risks (LRTI vs comparison) were 17.4 for CMC−, 6.9 for CMC+, and 6.8 for IC; corresponding associated risks were 2.8%, 5.0%, and 8.8%. At 360 days, relative risks were 3.9 for CMC−, 2.4 for CMC+, and 2.8 for IC; corresponding associated risks were 6.2%, 11.0%, and 20.3%. Mortality risk among hospitalized LRTI patients (and comparison patients) increased across comorbidity profiles within all age groups at each follow-up time point. Mortality risks among hospitalized LRTI patients (as well as ambulatory LRTI patients and matched comparison patients) using alternative age-stratification schemes are set forth in Supplementary Tables 3 and 4.

### Ambulatory LRTI

#### Patient Characteristics

Among the 5.6 million adults aged ≥18 years who received ambulatory care for LRTI during 2013–2018, 2.8 million met the minimum enrollment criterion (≥12 months pre-LRTI), and among these patients, 2.4 million met all remaining selection criteria and were matched to comparison patients. The mean (SD) age of ambulatory LRTI patients was 53.8 (18.3) years, and 35.6% of patients were designated as CMC+ (29.4%) or IC (6.2%); 21.5% of ambulatory LRTI patients had evidence of pneumonia.

#### Risk of Mortality

Risk of mortality among ambulatory LRTI patients was 1.2% (1.2%–1.2%) at 30 days, 2.6% (2.6%–2.6%) at 180 days, and 3.6% (3.6%–3.7%) at 360 days, 6.5, 2.8, and 2.1 times higher than the corresponding values for matched comparison patients (Table 3, Figure 1). LRTI-associated mortality risks were thus 1.0% at 30 days, 1.7% at 180 days, and 1.9% at 360 days. Risk of mortality among ambulatory LRTI patients increased with increasing age from 18–49 years to ≥85 years: from 0.1% (0.1%–0.1%) to 10.6% (10.4%–10.8%) at 30 days and from 0.2% (0.2%–0.2%) to 29.0% (28.7%–29.3%) at 360 days. Age-specific relative risks (vs comparison patients) ranged from 4.3 (≥85 years) to 8.9 (50–64 years) at 30 days and from 1.5 (≥85 years) to 2.8 (65–74 years) at 360 days; LRTI-associated risks ranged from 0.05% (18–49 years) to 8.1% (≥85 years) at 30 days and from 0.1% (18–49 years) to 9.7% (≥85 years) at 360 days.

**Table 3.** Risk of Mortality (All-Cause) Among Ambulatory LRTI Patients as Well as Matched Comparison Patients

		Risk of Mortality (95% CI), %														
	No. Matched Pairs	30 Days			60 Days			90 Days			180 Days			360 Days		
		LRTI Pts	Comp. Pts	RR	LRTI Pts	Comp. Pts	RR	LRTI Pts	Comp. Pts	RR	LRTI Pts	Comp. Pts	RR	LRTI Pts	Comp. Pts	RR
Overall	2 429 188	1.2 (1.2–1.2)	0.2 (0.2–0.2)	6.5 (6.3–6.7)	1.7 (1.7–1.7)	0.3 (0.3–0.4)	4.8 (4.7–4.9)	2.0 (1.9–2.0)	0.5 (0.5–0.5)	3.9 (3.8–4.0)	2.6 (2.6–2.6)	0.9 (0.9–1.0)	2.8 (2.8–2.8)	3.6 (3.6–3.7)	1.7 (1.7–1.7)	2.1 (2.1–2.2)
Age																
18–49 y	996 543	0.1 (0.1–0.1)	0.0 (0.0–0.0)	7.4 (5.9–9.3)	0.1 (0.1–0.1)	0.0 (0.0–0.0)	5.4 (4.5–6.4)	0.1 (0.1–0.1)	0.0 (0.0–0.0)	4.4 (3.8–5.0)	0.1 (0.1–0.2)	0.0 (0.0–0.1)	3.2 (2.9–3.6)	0.2 (0.2–0.2)	0.1 (0.1–0.1)	2.4 (2.2–2.6)
50–64 y	639 896	0.5 (0.5–0.5)	0.1 (0.1–0.1)	8.9 (8.0–9.9)	0.7 (0.7–0.7)	0.1 (0.1–0.1)	6.2 (5.7–6.7)	0.8 (0.8–0.9)	0.2 (0.2–0.2)	4.9 (4.6–5.2)	1.2 (1.1–1.2)	0.3 (0.3–0.4)	3.4 (3.3–3.6)	1.7 (1.6–1.7)	0.6 (0.6–0.7)	2.6 (2.5–2.7)
65–74 y	438 940	1.6 (1.6–1.7)	0.2 (0.2–0.2)	8.8 (8.2–9.5)	2.2 (2.2–2.3)	0.3 (0.3–0.4)	6.4 (6.0–6.7)	2.6 (2.6–2.7)	0.5 (0.5–0.5)	5.2 (4.9–5.4)	3.6 (3.5–3.6)	1.0 (0.9–1.0)	3.7 (3.6–3.9)	5.0 (4.9–5.1)	1.8 (1.8–1.8)	2.8 (2.7–2.9)
75–84 y	273 434	3.8 (3.7–3.8)	0.5 (0.5–0.5)	7.9 (7.4–8.3)	5.1 (5.1–5.2)	0.9 (0.9–0.9)	5.7 (5.4–5.9)	6.0 (5.9–6.1)	1.3 (1.3–1.3)	4.6 (4.5–4.8)	8.0 (7.9–8.1)	2.5 (2.4–2.5)	3.2 (3.1–3.3)	11.0 (10.9–11.2)	4.7 (4.6–4.8)	2.3 (2.3–2.4)
≥85 y	80 375	10.6 (10.4–10.8)	2.5 (2.4–2.6)	4.3 (4.1–4.5)	14.1 (13.9–14.4)	4.5 (4.3–4.6)	3.1 (3.0–3.3)	16.5 (16.2–16.8)	6.4 (6.3–6.6)	2.6 (2.5–2.6)	21.6 (21.4–21.9)	11.4 (11.2–11.7)	1.9 (1.8–1.9)	29.0 (28.7–29.3)	19.3 (19.0–19.5)	1.5 (1.5–1.5)
Comorbidity profile																
CMC−	1 564 418	0.3 (0.3–0.3)	0.0 (0.0–0.0)	11.8 (10.6–13.1)	0.4 (0.4–0.4)	0.0 (0.0–0.1)	8.0 (7.4–8.6)	0.4 (0.4–0.5)	0.1 (0.1–0.1)	6.1 (5.7–6.5)	0.6 (0.6–0.6)	0.1 (0.1–0.2)	4.1 (3.9–4.3)	0.8 (0.8–0.8)	0.3 (0.3–0.3)	2.8 (2.7–2.9)
CMC+	714 961	2.6 (2.6–2.6)	0.4 (0.4–0.4)	6.3 (6.0–6.5)	3.5 (3.5–3.6)	0.8 (0.8–0.8)	4.6 (4.4–4.7)	4.1 (4.1–4.2)	1.1 (1.1–1.1)	3.7 (3.6–3.8)	5.5 (5.5–5.6)	2.1 (2.1–2.1)	2.6 (2.6–2.7)	7.8 (7.7–7.8)	3.8 (3.8–3.9)	2.0 (2.0–2.1)
IC	149 809	4.5 (4.4–4.7)	0.8 (0.8–0.9)	5.6 (5.3–6.0)	6.4 (6.2–6.5)	1.5 (1.4–1.6)	4.2 (4.1–4.4)	7.5 (7.4–7.6)	2.1 (2.0–2.2)	3.6 (3.4–3.7)	9.9 (9.8–10.1)	3.7 (3.6–3.8)	2.7 (2.6–2.8)	13.3 (13.1–13.5)	6.2 (6.1–6.3)	2.1 (2.1–2.2)
Age and risk																
18–49 y																
CMC−	875 935	0.0 (0.0–0.0)	0.0 (0.0–0.0)	8.4 (5.8–12.1)	0.0 (0.0–0.0)	0.0 (0.0–0.0)	4.8 (3.7–6.1)	0.0 (0.0–0.1)	0.0 (0.0–0.0)	3.7 (3.0–4.6)	0.1 (0.1–0.1)	0.0 (0.0–0.0)	2.9 (2.5–3.3)	0.1 (0.1–0.1)	0.1 (0.0–0.1)	2.1 (1.9–2.3)
CMC+	102 017	0.2 (0.1–0.2)	0.0 (0.0–0.0)	5.4 (3.6–7.9)	0.2 (0.2–0.3)	0.0 (0.0–0.1)	4.5 (3.3–6.1)	0.3 (0.2–0.3)	0.1 (0.1–0.1)	4.1 (3.1–5.3)	0.4 (0.3–0.4)	0.1 (0.1–0.2)	2.9 (2.4–3.6)	0.6 (0.5–0.6)	0.2 (0.2–0.3)	2.4 (2.1–2.8)
IC	18 591	1.0 (0.9–1.2)	0.1 (0.1–0.2)	8.9 (5.7–14.0)	1.5 (1.3–1.7)	0.2 (0.1–0.3)	8.0 (5.6–11.5)	1.7 (1.5–1.9)	0.3 (0.2–0.4)	6.2 (4.6–8.2)	2.3 (2.1–2.6)	0.5 (0.4–0.7)	4.3 (3.5–5.4)	3.2 (2.9–3.4)	1.0 (0.9–1.2)	3.1 (2.7–3.7)
50–64 y																
CMC−	409 847	0.2 (0.2–0.2)	0.0 (0.0–0.0)	11.9 (9.0–15.6)	0.2 (0.2–0.2)	0.0 (0.0–0.0)	7.9 (6.5–9.6)	0.3 (0.3–0.3)	0.0 (0.0–0.1)	6.1 (5.2–7.2)	0.4 (0.3–0.4)	0.1 (0.1–0.1)	4.2 (3.7–4.7)	0.5 (0.5–0.6)	0.2 (0.2–0.2)	3.0 (2.7–3.2)
CMC+	188 214	0.8 (0.8–0.9)	0.1 (0.1–0.1)	8.3 (7.1–9.7)	1.1 (1.1–1.1)	0.2 (0.2–0.2)	5.7 (5.1–6.4)	1.3 (1.3–1.4)	0.3 (0.3–0.3)	4.7 (4.3–5.2)	1.8 (1.8–1.9)	0.6 (0.5–0.6)	3.3 (3.1–3.5)	2.7 (2.6–2.8)	1.1 (1.0–1.1)	2.5 (2.4–2.6)
IC	41 835	2.6 (2.5–2.8)	0.3 (0.3–0.4)	8.4 (7.0–10.1)	3.7 (3.5–3.8)	0.6 (0.5–0.7)	6.1 (5.3–7.0)	4.4 (4.2–4.6)	1.0 (0.9–1.1)	4.5 (4.1–5.1)	5.9 (5.6–6.1)	1.8 (1.7–1.9)	3.2 (3.0–3.5)	7.8 (7.6–8.1)	3.1 (3.0–3.3)	2.5 (2.4–2.7)
65–74 y																
CMC−	182 851	0.6 (0.5–0.6)	0.0 (0.0–0.0)	14.3 (11.2–18.1)	0.8 (0.7–0.8)	0.1 (0.1–0.1)	10.9 (9.1–13.0)	0.9 (0.8–0.9)	0.1 (0.1–0.1)	8.3 (7.2–9.7)	1.2 (1.2–1.3)	0.2 (0.2–0.2)	5.4 (4.8–6.0)	1.7 (1.7–1.8)	0.5 (0.4–0.5)	3.7 (3.4–4.0)
CMC+	210 100	1.8 (1.8–1.9)	0.2 (0.2–0.2)	10.5 (9.4–11.7)	2.5 (2.5–2.6)	0.4 (0.3–0.4)	6.8 (6.3–7.3)	3.0 (2.9–3.1)	0.6 (0.5–0.6)	5.4 (5.1–5.7)	4.1 (4.0–4.2)	1.1 (1.0–1.1)	3.8 (3.6–3.9)	5.9 (5.8–6.0)	2.1 (2.1–2.2)	2.7 (2.7–2.8)
IC	45 989	4.8 (4.6–5.0)	0.8 (0.7–0.9)	6.0 (5.4–6.7)	6.7 (6.5–6.9)	1.4 (1.3–1.5)	4.9 (4.5–5.4)	7.9 (7.6–8.1)	1.9 (1.8–2.0)	4.2 (3.9–4.5)	10.5 (10.3–10.8)	3.3 (3.1–3.4)	3.2 (3.0–3.4)	14.0 (13.7–14.3)	5.5 (5.3–5.7)	2.6 (2.5–2.7)
75–84 y																
CMC−	80 219	1.6 (1.5–1.7)	0.1 (0.1–0.1)	17.8 (14.1–22.6)	2.2 (2.1–2.3)	0.2 (0.2–0.2)	12.0 (10.2–14.2)	2.6 (2.5–2.7)	0.3 (0.2–0.3)	9.5 (8.3–10.9)	3.4 (3.3–3.5)	0.6 (0.5–0.6)	5.8 (5.3–6.4)	4.7 (4.6–4.8)	1.3 (1.3–1.4)	3.5 (3.3–3.7)
CMC+	158 476	4.2 (4.1–4.3)	0.5 (0.5–0.6)	8.1 (7.5–8.7)	5.7 (5.6–5.8)	1.0 (0.9–1.0)	5.9 (5.6–6.2)	6.7 (6.6–6.8)	1.4 (1.4–1.5)	4.7 (4.5–4.9)	8.9 (8.8–9.1)	2.8 (2.7–2.9)	3.2 (3.1–3.3)	12.5 (12.3–12.6)	5.4 (5.3–5.6)	2.3 (2.2–2.4)
IC	34 739	6.6 (6.3–6.9)	1.2 (1.1–1.3)	5.7 (5.1–6.3)	9.2 (8.9–9.5)	2.2 (2.1–2.4)	4.1 (3.8–4.4)	10.9 (10.5–11.2)	3.1 (2.9–3.3)	3.5 (3.3–3.8)	14.3 (13.9–14.7)	5.3 (5.1–5.5)	2.7 (2.6–2.8)	19.1 (18.7–19.5)	9.2 (8.9–9.5)	2.1 (2.0–2.2)
≥85 y																
CMC−	15 566	7.2 (6.8–7.6)	0.9 (0.8–1.0)	8.1 (6.8–9.7)	9.3 (8.9–9.8)	1.8 (1.6–2.0)	5.3 (4.6–6.0)	10.8 (10.3–11.3)	2.7 (2.5–3.0)	3.9 (3.5–4.4)	13.7 (13.2–14.3)	5.1 (4.8–5.5)	2.7 (2.5–2.9)	18.0 (17.4–18.6)	9.7 (9.2–10.2)	1.9 (1.8–2.0)
CMC+	56 154	11.3 (11.0–11.5)	2.8 (2.6–2.9)	4.1 (3.8–4.3)	15.0 (14.7–15.3)	4.9 (4.8–5.1)	3.0 (2.9–3.2)	17.6 (17.3–17.9)	7.1 (6.9–7.3)	2.5 (2.4–2.6)	23.3 (22.9–23.6)	12.6 (12.4–12.9)	1.8 (1.8–1.9)	31.3 (30.9–31.7)	21.2 (20.8–21.5)	1.5 (1.4–1.5)
IC	8655	12.2 (11.5–12.9)	3.4 (3.1–3.8)	3.5 (3.1–4.0)	16.8 (16.0–17.6)	6.4 (5.9–6.9)	2.6 (2.4–2.9)	19.7 (18.9–20.6)	8.8 (8.3–9.5)	2.2 (2.1–2.4)	25.5 (24.6–26.4)	15.1 (14.3–15.9)	1.7 (1.6–1.8)	34.0 (33.0–35.0)	24.0 (23.1–24.9)	1.4 (1.4–1.5)

Abbreviations: CI, confidence interval; CMC, chronic medical conditions; IC, immunocompromised; LRTI, lower respiratory tract infection; Pts, patients; RR, relative risk.

Mortality risks among ambulatory LRTI patients also increased with more complex comorbidity profiles: 0.3% (0.3%–0.3%) for CMC−, 2.6% (2.6%–2.6%) for CMC+, 4.5% (4.4%–4.7%) for IC at 30 days; 0.8% (0.8%–0.8%) for CMC−, 7.8% (7.7%–7.8%) for CMC+, 13.3% (13.1%–13.5%) for IC at 360 days. At 30 days, relative risks (LRTI vs comparison) were 11.8 for CMC−, 6.3 for CMC+, and 5.6 for IC; corresponding associated risks were 0.3%, 2.2%, and 3.7%. At 360 days, relative risks were 2.8 for CMC−, 2.0 for CMC+, and 2.1 for IC; corresponding associated risks were 0.5%, 3.9%, and 7.1%. Mortality risk among ambulatory LRTI patients (and comparison patients) increased across comorbidity profiles within all age groups at each follow-up time point. Relative risks were often highest for CMC− patients, both overall and across age groups, due to low mortality risk among comparison patients.

## DISCUSSION

In this retrospective study including >60 000 hospitalized LRTI patients and 2.4 million ambulatory LRTI patients, we evaluated short- and long-term mortality risks among adults of all ages and various comorbidity profiles. To the best of our knowledge, this study is the first to evaluate the risk of mortality following LRTI across subgroups defined on age and comorbidity profile using a matched-cohort design and a large US health care claims database. Study findings indicate that LRTI patients (both hospitalized and ambulatory) have considerably higher mortality risks than their matched (non-LRTI) comparison patients. While relative risks decreased over time, differences between LRTI patients and matched comparison patients persisted. Moreover, LRTI-associated risks increased with longer follow-up periods.

The study findings also indicate that mortality risk increases markedly with increasing age at each follow-up time point and that mortality risk is substantially higher among LRTI patients with chronic medical conditions or immunocompromising conditions vs their healthy counterparts. While, as expected, mortality risks were lower among ambulatory (vs hospitalized) LRTI patients, risks increased with age and the presence of comorbidities. Additionally, similar to the overall findings, age-specific relative risks decreased but persisted over time while LRTI-associated risks were relatively constant. Such findings were consistent within subgroups defined on age and comorbidity profile. In addition, although mortality increased with age across all subgroups, younger LRTI patients with comorbidities had—in many instances—mortality risks that were comparable to or greater than those among older LRTI patients without comorbidities.

Notwithstanding differences in study designs and study populations, the mortality risks for hospitalized LRTI reported herein are, with few exceptions, largely consistent with those from previously published research. In a 2021 retrospective study evaluating all-cause mortality in the year following hospitalization for pneumonia among US adults (n = 37 006; mean age, 71 years), mortality following admission was reported to be 8.2% at 30 days (vs 5.8% in our study) and 17.7% at 360 days (vs 18.3%) and was reported to increase with increasing age and worsening comorbidity profile [7]. Differences in mortality risks at earlier follow-up time points may be attributed to our focus on all LRTIs requiring hospitalization, vs pneumonia in the study by Averin and colleagues. In a study of Medicare fee-for-service beneficiaries (2007–2008), 30-day mortality among patients hospitalized for pneumonia was reported to be 8.5% (vs 7.3% among patients aged ≥65 years in our study), and risk of early mortality increased with age and increasing severity of comorbidity profile [12].

In a more recent analysis of Medicare fee-for-service patients aged ≥65 years (2008–2016), 30-day postdischarge mortality was 8.4% [13]. Finally, in a prospective population-based study of hospitalized pneumonia patients (n = 7449; median age, 68 years) in Louisville, Kentucky, mortality was 13.1% at 30 days, 23.4% at 6 months postdischarge, and 30.6% at 12 months postdischarge [6]. The higher mortality risks in the Louisville study are likely due to differences in study design, as Ramirez et al. employed a prospective study design and strict inclusion criteria (eg, chest exams, symptoms, lack of alternative diagnosis), whereas the present study was retrospective and relied on diagnosis codes for selection of LRTI patients for the study population.

We also note that our study is one of the first to report short-term and long-term mortality risks among adults with ambulatory LRTI and that our findings are comparable to the limited data available from published literature. For example, in a 2015 article, Eurich et al. report findings from a study of long-term mortality among Canadian adults with pneumonia requiring hospitalization (n = 2653) or emergency department care (n = 3425) vs matched comparison patients [14]. In this study, rates of all-cause mortality for ambulatory pneumonia patients and comparison patients were 36 and 22, respectively, per 1000 person-years, equivalent to 3.6% and 2.2% mortality (vs 3.6% and 1.7% in our study). Additional data on mortality by age and/or comorbidity profile among ambulatory pneumonia patients were not reported in the Eurich et al. publication.

While the Optum CDM provides information on large numbers of patients with specific diagnoses who receive care for specific conditions, several limitations from its use in our study should be noted. Although the algorithm for identifying LRTI has been used in prior studies, to the best of our knowledge, this algorithm has not been formally evaluated against a “gold standard” and thus its accuracy (ie, in terms of sensitivity and specificity) is unknown [15, 16]. In our study, the proportion of LRTI manifested as pneumonia was 90% for hospitalized episodes (22% for ambulatory episodes), which is somewhat higher than findings from other studies (range, 67%–79%) [17–19]. Differences may be attributable to study design, data sources, case ascertainment criteria, and composition of study populations (eg, by age, comorbidity profile).

Similarly, use of operational algorithms to characterize comorbidity profiles undoubtedly resulted in misclassification of some adults who have the underlying conditions as well as some adults who do not have the underlying conditions. We note that these algorithms have been employed in several previously published studies [20–24]. We also note that although comorbidity profiles were defined based on medical conditions listed in the US ACIP recommendations for influenza vaccination [11], high-risk conditions listed by the US ACIP for other adult vaccinations (eg, RSV) are comparable; accordingly, we believe that the classification scheme employed is generalizable to LRTI as a whole [25]. We also note that we did not employ time-dependent variables to identify the presence of new comorbidities during the follow-up period (ie, comorbidities not present at baseline) due to uncertainty regarding the specific dates of onset/diagnoses for the conditions of interest. We believe, however, that given the relatively short follow-up period, the impact of this limitation on misclassification of adults by comorbidity profile was minimal.

Because comparison patients were matched to LRTI patients based on age, sex, comorbidity profile, and health care profile, their mortality risks (especially for those matched to hospitalized LRTI patients) are likely different from those among the general US population. While comparison patients matched to hospitalized LRTI patients were not required to have evidence of hospitalization, they were matched on other factors (eg, age, comorbidity profile, health care profile) that could confound study results. However, we acknowledge that associated risks may be inflated somewhat. In addition, LRTI patients and matched comparison patients may differ based on unobserved characteristics, and thus the possibility exists that differences in mortality risks between LRTI patients and comparison patients may be associated with, at least in part, differences in unobserved characteristics. For example, hospitalized LRTI patients may have worse health status that increases the risk of mortality at baseline compared with comparison patients. While it was anticipated that the Optum CDM would be sufficiently large to evaluate study objectives among adults within age-specific subgroups, evaluations within subgroups defined on age and comorbidity profile may lack adequate precision and should be interpreted with caution. Finally, while the Optum CDM includes information on many patients across demographic profiles, providers, and geographic regions, caution should be employed in generalizing study results to other populations and settings.

In conclusion, the findings from this study indicate that LRTI patients, both hospitalized and ambulatory, have elevated mortality risks compared with matched comparison patients in the short term and long term, especially those of older ages and with worse comorbidity profiles (irrespective of age). Implementing strategies to prevent LRTI in adults of all ages has the potential to yield important public health and patient benefits.

## Supplementary Material

ofaf186_Supplementary_Data

## Supplementary Data

Supplementary materials are available at *Open Forum Infectious Diseases* online. Consisting of data provided by the authors to benefit the reader, the posted materials are not copyedited and are the sole responsibility of the authors, so questions or comments should be addressed to the corresponding author.
