# Supplementary material for: Short-term and Long-term Mortality Following Hospitalized and Ambulatory Lower Respiratory Tract Illnesses Among US Adults
Source: Open Forum Infect Dis. 2025 Mar 26;12(4):ofaf186. doi: 10.1093/ofid/ofaf186 (PMC12000873; doi:10.1093/ofid/ofaf186)
Supplement: ofaf186_Supplementary_Data [file ofaf186_supplementary_data.pdf]

Supplement -- Table 1. Baseline characteristics of hospitalized LRTI patients (and matched comparison patients*)

		Age (years)					Risk Profile		
	Overall (N=60,208)	18-49 (N=5,211)	50-64 (N=11,147)	65-74 (N=15,478)	75-84 (N=18,100)	≥85 (N=10,272)	CMC- (N=15,495)	CMC+ (N=35,207)	IC (N=9,506)
Demographic Profile									
Age (years), mean (SD)	71.0 (14.4)	38.5 (8.7)	58.1 (4.1)	69.9 (2.7)	79.8 (3.1)	87.3 (1.8)	64.2 (17.7)	73.8 (12.1)	71.2 (12.1)
Age groups (years), %									
18-49	8.7	100.0	0.0	0.0	0.0	0.0	21.0	4.1	5.2
50-64	18.5	0.0	100.0	0.0	0.0	0.0	23.9	15.6	20.5
65-74	25.7	0.0	0.0	100.0	0.0	0.0	21.2	26.5	30.1
75-84	30.1	0.0	0.0	0.0	100.0	0.0	21.7	33.1	32.5
≥85	17.1	0.0	0.0	0.0	0.0	100.0	12.1	20.7	11.7
Sex, %									
Male	45.5	49.2	49.3	48.2	45.0	36.1	44.7	45.2	47.9
Female	54.5	50.8	50.7	51.8	55.0	63.9	55.3	54.8	52.1
Comorbidity Profile, %									
CMC-	25.7	62.6	33.2	21.2	18.6	18.3	100.0	0.0	0.0
CMC+									
Cardiopulmonary	0.9	0.1	0.5	0.9	1.1	1.4	0.0	1.3	0.9
Cardiovascular	39.3	5.3	24.3	39.1	49.1	55.8	0.0	55.7	42.6
Hematologic	6.4	3.2	5.7	7.1	7.4	6.2	0.0	6.7	15.9
Hepatic	0.8	0.3	1.9	1.3	0.4	0.1	0.0	1.1	1.2
Metabolic	31.5	12.6	30.8	38.9	34.5	25.3	0.0	45.6	30.2
Neurologic	19.8	4.5	9.7	15.7	24.8	36.1	0.0	29.3	17.0
Pulmonary	24.9	12.1	24.9	31.2	25.6	20.6	0.0	35.1	27.8
Renal	15.3	2.7	8.6	14.6	19.8	22.4	0.0	21.3	18.4
Obesity (BMI >40)	3.4	4.8	6.6	4.8	1.6	0.3	0.0	5.3	2.0
IC	15.8	9.5	17.5	18.5	17.1	10.9	0.0	0.0	100.0
Healthcare Profile									
No. all-cause ambulatory encounters, %									
0	1.9	4.7	2.6	1.9	1.2	1.1	7.5	0.0	0.0
1-9	18.8	41.1	22.8	17.1	14.2	13.7	46.3	10.8	3.4
10-19	22.3	22.3	22.3	21.9	22.8	21.8	25.8	23.5	12.0
≥20	57.0	31.8	52.3	59.1	61.8	63.4	20.3	65.7	84.6
No. all-cause hospitalizations, %									
0	74.0	82.9	75.4	73.4	73.4	70.0	94.0	69.0	59.9
1	13.8	11.2	14.0	14.6	13.9	13.3	4.7	16.1	20.1
≥2	12.2	5.8	10.6	12.0	12.7	16.7	1.3	14.9	20.0
Healthcare costs ($), %									
<$1,000	11.0	22.4	13.3	10.0	8.4	8.6	31.9	4.4	1.1
$1,000-$4,999	25.2	30.5	24.4	23.9	25.3	24.9	35.4	24.4	11.6
$5,000-$9,999	14.8	13.7	14.5	14.7	14.9	15.8	14.4	16.1	10.6
$10,000-$19,999	14.4	11.3	13.0	14.3	15.7	15.7	9.5	17.2	12.4
$20,000-$49,999	16.7	11.4	15.3	16.1	18.0	19.2	6.5	20.1	20.4
≥$50,000	17.9	10.7	19.5	21.0	17.6	15.7	2.3	17.8	43.8
Duration of follow-up, mean (SD)	1,037 (878)	946 (873)	972 (854)	1,118 (896)	1,220 (947)	710 (600)	1,145 (901)	1,034 (862)	873 (871)

LRTI: lower respiratory tract infection; CMC: chronic medical conditions; IC: immunocompromised

*LRTI and comparison patients were matched 1:1 on baseline characteristics, thus means and percentages are identical between subgroups

Supplement -- Table 2. Baseline characteristics of ambulatory LRTI patients (and matched comparison patients*)

		Age (years)					Risk Profile		
	Overall (N=2,429,188)	18-49 (N=996,543)	50-64 (N=639,896)	65-74 (N=438,940)	75-84 (N=273,434)	≥85 (N=80,375)	CMC- (N=1,564,418)	CMC+ (N=714,961)	IC (N=149,809)
Demographic Profile									
Age (years), mean (SD)	53.8 (18.3)	35.4 (9.1)	56.8 (4.2)	69.5 (2.7)	79.2 (3.1)	87.0 (1.8)	47.3 (16.8)	65.6 (14.8)	65.9 (13.6)
Age groups (years), %									
18-49	41.0	100.0	0.0	0.0	0.0	0.0	56.0	14.3	12.4
50-64	26.3	0.0	100.0	0.0	0.0	0.0	26.2	26.3	27.9
65-74	18.1	0.0	0.0	100.0	0.0	0.0	11.7	29.4	30.7
75-84	11.3	0.0	0.0	0.0	100.0	0.0	5.1	22.2	23.2
≥85	3.3	0.0	0.0	0.0	0.0	100.0	1.0	7.9	5.8
Sex, %									
Male	43.8	44.3	45.5	43.3	41.9	33.9	43.8	44.1	42.4
Female	56.2	55.7	54.5	56.7	58.1	66.1	56.2	55.9	57.6
Comorbidity Profile, %									
CMC-	64.4	87.9	64.0	41.7	29.3	19.4	100.0	0.0	0.0
CMC+									
Cardiopulmonary	0.2	0.0	0.1	0.3	0.7	1.3	0.0	0.6	0.4
Cardiovascular	13.5	0.9	9.4	24.4	39.6	54.2	0.0	40.1	27.7
Hematologic	1.6	0.3	1.2	2.7	4.2	5.2	0.0	3.7	8.3
Hepatic	0.5	0.1	0.9	0.9	0.4	0.1	0.0	1.4	1.2
Metabolic	15.5	4.1	17.0	28.6	29.6	25.4	0.0	47.6	24.4
Neurologic	5.9	1.0	3.5	8.2	17.3	35.4	0.0	18.0	10.3
Pulmonary	10.2	3.8	10.1	17.7	19.2	20.3	0.0	30.9	18.9
Renal	4.6	0.3	2.3	7.9	15.2	22.2	0.0	13.3	11.3
Obesity (BMI >40)	2.2	1.6	3.0	3.2	1.3	0.3	0.0	7.0	2.1
IC	6.2	1.9	6.5	10.5	12.7	10.8	0.0	0.0	100.0
Healthcare Profile									
No. all-cause ambulatory encounters, %									
0	5.2	9.0	4.1	1.7	1.1	0.8	8.2	0.0	0.0
1-9	40.8	55.0	40.8	26.8	19.2	14.4	54.6	17.7	7.4
10-19	25.1	21.0	26.9	30.2	28.3	22.3	22.9	30.5	22.1
≥20	28.9	14.9	28.2	41.3	51.3	62.4	14.4	51.8	70.5
No. all-cause hospitalizations, %									
0	90.5	95.0	92.2	87.0	81.8	71.4	96.5	80.6	75.3
1	6.5	4.3	5.6	8.6	10.7	14.0	3.1	12.2	14.4
≥2	3.0	0.7	2.2	4.4	7.5	14.7	0.4	7.1	10.3
Healthcare costs ($), %									
<$1,000	30.3	45.1	27.7	15.8	11.7	9.0	42.9	8.5	3.0
$1,000-$4,999	34.3	33.2	36.5	35.8	32.7	27.6	35.9	33.0	23.1
$5,000-$9,999	12.8	9.3	13.6	16.4	16.8	16.0	10.3	17.5	15.5
$10,000-$19,999	9.6	6.8	9.5	12.5	14.1	15.1	6.4	15.6	14.7
$20,000-$49,999	7.8	4.2	7.8	10.8	13.3	17.6	3.6	15.0	18.4
≥$50,000	5.2	1.4	4.9	8.8	11.4	14.7	0.9	10.5	25.4
Duration of follow-up, mean (SD)	1,140 (899)	991 (855)	1,101 (885)	1,386 (916)	1,475 (951)	812 (624)	1,111 (891)	1,199 (906)	1,158 (944)

LRTI: lower respiratory tract infection; CMC: chronic medical conditions; IC: immunocompromised

*LRTI and comparison patients were matched 1:1 on baseline characteristics, thus means and percentages are identical between subgroups

Supplement -- Table 3. Risk of mortality (all-cause) among hospitalized LRTI patients as well as matched comparison patients using alternative age stratification schemes

		Risk of Mortality, % (95% CI)														
		30 Day			60 Day			90 Day			180 Day			360 Day		
	No. Matched Pairs	LRTI Pts	Comp. Pts	RR	LRTI Pts	Comp. Pts	RR	LRTI Pts	Comp. Pts	RR	LRTI Pts	Comp. Pts	RR	LRTI Pts	Comp. Pts	RR
Overall	60,208	5.8 (5.7-6.0)	0.8 (0.7-0.9)	7.5 (6.8-8.2)	8.5 (8.3-8.7)	1.6 (1.5-1.7)	5.4 (5.0-5.8)	10.1 (9.9-10.4)	2.2 (2.1-2.3)	4.6 (4.3-4.8)	13.5 (13.3-13.8)	4.1 (3.9-4.2)	3.3 (3.2-3.5)	18.3 (18.0-18.6)	7.1 (6.9-7.3)	2.6 (2.5-2.7)
Age (years)																
18-59	11,682	1.6 (1.4-1.9)	0.1 (0.1-0.2)	11.2 (6.8-18.3)	2.5 (2.2-2.8)	0.3 (0.2-0.4)	8.5 (5.9-12.1)	3.0 (2.7-3.3)	0.4 (0.3-0.5)	7.7 (5.6-10.5)	4.1 (3.8-4.5)	0.7 (0.6-0.9)	5.8 (4.6-7.3)	5.5 (5.1-6.0)	1.2 (1.0-1.4)	4.5 (3.8-5.4)
60-64	4,676	3.1 (2.7-3.7)	0.3 (0.2-0.6)	9.2 (5.5-15.4)	4.8 (4.2-5.4)	0.8 (0.6-1.1)	5.7 (4.1-8.0)	5.7 (5.1-6.4)	1.0 (0.8-1.4)	5.6 (4.1-7.5)	8.1 (7.3-8.9)	1.8 (1.4-2.2)	4.6 (3.6-5.8)	11.2 (10.4-12.2)	3.0 (2.6-3.5)	3.7 (3.1-4.5)
≥65	43,850	7.3 (7.0-7.5)	1.0 (0.9-1.1)	7.2 (6.6-8.0)	10.5 (10.2-10.8)	2.0 (1.9-2.1)	5.3 (4.9-5.6)	12.5 (12.2-12.8)	2.8 (2.7-3.0)	4.4 (4.1-4.7)	16.6 (16.3-17.0)	5.2 (5.0-5.4)	3.2 (3.1-3.4)	22.4 (22.1-22.8)	9.0 (8.8-9.3)	2.5 (2.4-2.6)
<60	11,682	1.6 (1.4-1.9)	0.1 (0.1-0.2)	11.2 (6.8-18.3)	2.5 (2.2-2.8)	0.3 (0.2-0.4)	8.5 (5.9-12.1)	3.0 (2.7-3.3)	0.4 (0.3-0.5)	7.7 (5.6-10.5)	4.1 (3.8-4.5)	0.7 (0.6-0.9)	5.8 (4.6-7.3)	5.5 (5.1-6.0)	1.2 (1.0-1.4)	4.5 (3.8-5.4)
≥60	48,526	6.9 (6.6-7.1)	0.9 (0.9-1.0)	7.3 (6.6-8.1)	10.0 (9.7-10.2)	1.9 (1.8-2.0)	5.3 (4.9-5.7)	11.9 (11.6-12.2)	2.7 (2.5-2.8)	4.4 (4.2-4.7)	15.8 (15.5-16.1)	4.9 (4.7-5.1)	3.3 (3.1-3.4)	21.4 (21.0-21.7)	8.5 (8.2-8.7)	2.5 (2.4-2.6)
Comorbidity profile																
CMC-	15,495	2.9 (2.7-3.2)	0.2 (0.1-0.2)	17.4 (11.7-25.9)	4.1 (3.8-4.5)	0.4 (0.3-0.5)	11.3 (8.6-14.8)	4.9 (4.5-5.2)	0.6 (0.5-0.8)	7.7 (6.2-9.5)	6.3 (6.0-6.7)	1.2 (1.0-1.4)	5.4 (4.6-6.3)	8.3 (7.9-8.7)	2.1 (1.9-2.3)	3.9 (3.5-4.4)
CMC+	35,207	5.9 (5.7-6.2)	0.9 (0.8-1.0)	6.9 (6.1-7.8)	8.5 (8.3-8.8)	1.7 (1.6-1.8)	5.0 (4.6-5.5)	10.2 (9.9-10.5)	2.4 (2.2-2.6)	4.3 (4.0-4.6)	13.8 (13.4-14.1)	4.5 (4.3-4.8)	3.0 (2.9-3.2)	19.1 (18.7-19.5)	8.1 (7.8-8.4)	2.4 (2.3-2.5)
IC	9,506	10.4 (9.8-11.0)	1.5 (1.3-1.8)	6.8 (5.7-8.1)	15.5 (14.8-16.2)	3.1 (2.8-3.5)	5.0 (4.4-5.6)	18.4 (17.7-19.2)	4.2 (3.8-4.6)	4.4 (4.0-4.9)	24.4 (23.6-25.3)	7.0 (6.5-7.5)	3.5 (3.2-3.8)	31.6 (30.7-32.5)	11.3 (10.7-12.0)	2.8 (2.6-3.0)
Age (years) and risk																
18-59																
CMC-	5,753	0.7 (0.5-1.0)	0.0 (0.0-0.1)	20.5 (5.0-84.7)	1.0 (0.8-1.3)	0.0 (0.0-0.1)	29.0 (7.1-118.7)	1.1 (0.9-1.5)	0.0 (0.0-0.1)	33.0 (8.1-134.7)	1.5 (1.3-1.9)	0.0 (0.0-0.1)	44.5 (11.0-180.6)	1.9 (1.6-2.3)	0.1 (0.0-0.2)	22.4 (9.2-54.8)
CMC+	4,396	1.5 (1.1-1.9)	0.2 (0.1-0.3)	9.1 (4.2-19.9)	2.2 (1.8-2.7)	0.3 (0.2-0.6)	6.4 (3.7-11.0)	2.7 (2.2-3.2)	0.4 (0.3-0.7)	6.2 (3.8-10.0)	3.8 (3.3-4.4)	0.8 (0.6-1.1)	4.7 (3.3-6.8)	5.5 (4.8-6.2)	1.5 (1.2-1.9)	3.6 (2.7-4.7)
IC	1,533	5.5 (4.5-6.8)	0.5 (0.3-1.0)	10.6 (5.2-21.9)	8.7 (7.4-10.3)	1.1 (0.7-1.8)	7.9 (4.8-13.0)	10.6 (9.2-12.3)	1.6 (1.1-2.3)	6.8 (4.5-10.4)	14.7 (13.1-16.6)	3.0 (2.3-4.0)	4.9 (3.6-6.7)	19.2 (17.4-21.3)	4.7 (3.7-5.9)	4.1 (3.2-5.2)
60-64																
CMC-	1,209	1.7 (1.1-2.6)	0.1 (0.0-0.6)	20.0 (2.7-148.8)	2.3 (1.6-3.3)	0.2 (0.1-0.8)	9.3 (2.8-30.6)	2.9 (2.1-4.0)	0.2 (0.1-0.8)	11.7 (3.6-37.8)	3.6 (2.7-4.8)	0.5 (0.2-1.1)	7.2 (3.1-16.8)	4.3 (3.3-5.6)	0.6 (0.3-1.2)	7.4 (3.4-16.3)
CMC+	2,557	2.2 (1.7-2.8)	0.2 (0.1-0.5)	11.2 (4.5-27.9)	3.4 (2.7-4.1)	0.6 (0.4-1.0)	5.7 (3.3-9.9)	3.9 (3.3-4.8)	0.8 (0.5-1.3)	4.8 (3.0-7.7)	6.0 (5.1-7.0)	1.4 (1.1-2.0)	4.1 (2.9-5.9)	9.2 (8.2-10.4)	2.9 (2.3-3.6)	3.2 (2.5-4.1)
IC	910	7.8 (6.2-9.8)	1.1 (0.6-2.0)	7.1 (3.7-13.7)	12.0 (10.0-14.3)	2.3 (1.5-3.5)	5.2 (3.3-8.2)	14.4 (12.3-16.9)	2.6 (1.8-3.9)	5.5 (3.6-8.4)	20.0 (17.6-22.8)	4.4 (3.2-6.0)	4.5 (3.3-6.3)	26.2 (23.4-29.2)	6.6 (5.2-8.4)	4.0 (3.0-5.2)
≥65																
CMC-	8,533	4.6 (4.2-5.1)	0.3 (0.2-0.4)	17.0 (11.2-25.9)	6.5 (6.0-7.1)	0.6 (0.5-0.8)	10.7 (8.1-14.2)	7.6 (7.1-8.2)	1.1 (0.9-1.3)	7.0 (5.6-8.7)	9.9 (9.3-10.6)	2.0 (1.8-2.4)	4.9 (4.2-5.7)	13.1 (12.4-13.8)	3.7 (3.3-4.1)	3.5 (3.1-4.0)
CMC+	28,254	6.9 (6.6-7.2)	1.0 (0.9-1.1)	6.8 (6.0-7.7)	10.0 (9.7-10.4)	2.0 (1.9-2.2)	5.0 (4.6-5.4)	12.0 (11.6-12.4)	2.8 (2.7-3.0)	4.2 (3.9-4.5)	16.0 (15.6-16.5)	5.4 (5.1-5.7)	3.0 (2.8-3.1)	22.1 (21.7-22.6)	9.6 (9.2-9.9)	2.3 (2.2-2.4)
IC	7,063	11.8 (11.0-12.5)	1.8 (1.5-2.1)	6.5 (5.4-7.9)	17.4 (16.5-18.3)	3.6 (3.2-4.1)	4.8 (4.2-5.4)	20.6 (19.7-21.6)	4.9 (4.5-5.5)	4.2 (3.7-4.7)	27.1 (26.1-28.2)	8.2 (7.6-8.8)	3.3 (3.0-3.6)	35.0 (33.9-36.1)	13.4 (12.6-14.2)	2.6 (2.4-2.8)
<60																
CMC-	5,753	0.7 (0.5-1.0)	0.0 (0.0-0.1)	20.5 (5.0-84.7)	1.0 (0.8-1.3)	0.0 (0.0-0.1)	29.0 (7.1-118.7)	1.1 (0.9-1.5)	0.0 (0.0-0.1)	33.0 (8.1-134.7)	1.5 (1.3-1.9)	0.0 (0.0-0.1)	44.5 (11.0-180.6)	1.9 (1.6-2.3)	0.1 (0.0-0.2)	22.4 (9.2-54.8)
CMC+	4,396	1.5 (1.1-1.9)	0.2 (0.1-0.3)	9.1 (4.2-19.9)	2.2 (1.8-2.7)	0.3 (0.2-0.6)	6.4 (3.7-11.0)	2.7 (2.2-3.2)	0.4 (0.3-0.7)	6.2 (3.8-10.0)	3.8 (3.3-4.4)	0.8 (0.6-1.1)	4.7 (3.3-6.8)	5.5 (4.8-6.2)	1.5 (1.2-1.9)	3.6 (2.7-4.7)
IC	1,533	5.5 (4.5-6.8)	0.5 (0.3-1.0)	10.6 (5.2-21.9)	8.7 (7.4-10.3)	1.1 (0.7-1.8)	7.9 (4.8-13.0)	10.6 (9.2-12.3)	1.6 (1.1-2.3)	6.8 (4.5-10.4)	14.7 (13.1-16.6)	3.0 (2.3-4.0)	4.9 (3.6-6.7)	19.2 (17.4-21.3)	4.7 (3.7-5.9)	4.1 (3.2-5.2)
≥60																
CMC-	9,742	4.2 (3.8-4.6)	0.2 (0.2-0.4)	17.2 (11.4-25.9)	6.0 (5.6-6.5)	0.6 (0.4-0.7)	10.6 (8.1-14.0)	7.0 (6.6-7.6)	1.0 (0.8-1.2)	7.1 (5.8-8.8)	9.2 (8.6-9.7)	1.8 (1.6-2.1)	5.0 (4.2-5.8)	12.0 (11.4-12.7)	3.3 (3.0-3.7)	3.6 (3.2-4.1)
CMC+	30,811	6.5 (6.3-6.8)	1.0 (0.9-1.1)	6.9 (6.1-7.7)	9.5 (9.1-9.8)	1.9 (1.7-2.1)	5.0 (4.6-5.5)	11.3 (11.0-11.7)	2.7 (2.5-2.9)	4.2 (3.9-4.5)	15.2 (14.8-15.6)	5.1 (4.8-5.3)	3.0 (2.8-3.2)	21.1 (20.6-21.5)	9.0 (8.7-9.3)	2.3 (2.2-2.4)
IC	7,973	11.3 (10.6-12.0)	1.7 (1.5-2.0)	6.6 (5.5-7.9)	16.8 (16.0-17.6)	3.5 (3.1-3.9)	4.8 (4.2-5.4)	19.9 (19.1-20.8)	4.7 (4.2-5.2)	4.3 (3.8-4.7)	26.3 (25.4-27.3)	7.7 (7.2-8.3)	3.4 (3.1-3.7)	34.0 (32.9-35.0)	12.6 (11.9-13.4)	2.7 (2.5-2.9)

LRTI: lower respiratory tract infection; RR: relative risk; CMC: chronic medical conditions; IC: immunocompromised

Supplement -- Table 4. Risk of mortality (all-cause) among ambulatory LRTI patients as well as matched comparison patients using alternative age stratification schemes

	No. Matched Pairs	Risk of Mortality, % (95% CI)														
		30 Day			60 Day			90 Day			180 Day			360 Day		
		LRTI Pts	Comp. Pts	RR	LRTI Pts	Comp. Pts	RR	LRTI Pts	Comp. Pts	RR	LRTI Pts	Comp. Pts	RR	LRTI Pts	Comp. Pts	RR
Overall	2,429,188	1.2 (1.2-1.2)	0.2 (0.2-0.2)	6.5 (6.3-6.7)	1.7 (1.7-1.7)	0.3 (0.3-0.4)	4.8 (4.7-4.9)	2.0 (1.9-2.0)	0.5 (0.5-0.5)	3.9 (3.8-4.0)	2.6 (2.6-2.6)	0.9 (0.9-1.0)	2.8 (2.8-2.8)	3.6 (3.6-3.7)	1.7 (1.7-1.7)	2.1 (2.1-2.2)
Age (years)																
18-59	1,439,758	0.2 (0.2-0.2)	0.0 (0.0-0.0)	7.9 (7.0-9.0)	0.2 (0.2-0.2)	0.0 (0.0-0.0)	5.7 (5.2-6.2)	0.3 (0.3-0.3)	0.1 (0.1-0.1)	4.5 (4.2-4.9)	0.4 (0.4-0.4)	0.1 (0.1-0.1)	3.3 (3.1-3.5)	0.5 (0.5-0.5)	0.2 (0.2-0.2)	2.5 (2.4-2.6)
60-64	196,681	0.8 (0.8-0.9)	0.1 (0.1-0.1)	9.8 (8.3-11.4)	1.1 (1.1-1.2)	0.2 (0.2-0.2)	6.6 (5.9-7.4)	1.4 (1.3-1.4)	0.3 (0.2-0.3)	5.2 (4.7-5.7)	1.8 (1.8-1.9)	0.5 (0.5-0.6)	3.5 (3.3-3.8)	2.6 (2.6-2.7)	1.0 (1.0-1.1)	2.6 (2.5-2.7)
≥65	792,749	3.3 (3.2-3.3)	0.5 (0.5-0.5)	6.3 (6.1-6.5)	4.4 (4.4-4.5)	1.0 (0.9-1.0)	4.6 (4.5-4.7)	5.2 (5.2-5.3)	1.4 (1.4-1.4)	3.8 (3.7-3.8)	6.9 (6.9-7.0)	2.5 (2.5-2.6)	2.7 (2.7-2.8)	9.5 (9.5-9.6)	4.6 (4.5-4.6)	2.1 (2.1-2.1)
<60	1,439,758	0.2 (0.2-0.2)	0.0 (0.0-0.0)	7.9 (7.0-9.0)	0.2 (0.2-0.2)	0.0 (0.0-0.0)	5.7 (5.2-6.2)	0.3 (0.3-0.3)	0.1 (0.1-0.1)	4.5 (4.2-4.9)	0.4 (0.4-0.4)	0.1 (0.1-0.1)	3.3 (3.1-3.5)	0.5 (0.5-0.5)	0.2 (0.2-0.2)	2.5 (2.4-2.6)
≥60	989,430	2.8 (2.7-2.8)	0.4 (0.4-0.4)	6.4 (6.2-6.7)	3.8 (3.7-3.8)	0.8 (0.8-0.8)	4.7 (4.6-4.8)	4.4 (4.4-4.5)	1.2 (1.1-1.2)	3.8 (3.8-3.9)	5.9 (5.9-6.0)	2.1 (2.1-2.2)	2.8 (2.7-2.8)	8.2 (8.1-8.2)	3.9 (3.8-3.9)	2.1 (2.1-2.1)
Comorbidity profile																
CMC-	1,564,418	0.3 (0.3-0.3)	0.0 (0.0-0.0)	11.8 (10.6-13.1)	0.4 (0.4-0.4)	0.0 (0.0-0.1)	8.0 (7.4-8.6)	0.4 (0.4-0.5)	0.1 (0.1-0.1)	6.1 (5.7-6.5)	0.6 (0.6-0.6)	0.1 (0.1-0.2)	4.1 (3.9-4.3)	0.8 (0.8-0.8)	0.3 (0.3-0.3)	2.8 (2.7-2.9)
CMC+	714,961	2.6 (2.6-2.6)	0.4 (0.4-0.4)	6.3 (6.0-6.5)	3.5 (3.5-3.6)	0.8 (0.8-0.8)	4.6 (4.4-4.7)	4.1 (4.1-4.2)	1.1 (1.1-1.1)	3.7 (3.6-3.8)	5.5 (5.5-5.6)	2.1 (2.1-2.1)	2.6 (2.6-2.7)	7.8 (7.7-7.8)	3.8 (3.8-3.9)	2.0 (2.0-2.1)
IC	149,809	4.5 (4.4-4.7)	0.8 (0.8-0.9)	5.6 (5.3-6.0)	6.4 (6.2-6.5)	1.5 (1.4-1.6)	4.2 (4.1-4.4)	7.5 (7.4-7.6)	2.1 (2.0-2.2)	3.6 (3.4-3.7)	9.9 (9.8-10.1)	3.7 (3.6-3.8)	2.7 (2.6-2.8)	13.3 (13.1-13.5)	6.2 (6.1-6.3)	2.1 (2.1-2.2)
Age (years) and risk																
18-59																
CMC-	1,179,066	0.1 (0.1-0.1)	0.0 (0.0-0.0)	9.6 (7.5-12.2)	0.1 (0.1-0.1)	0.0 (0.0-0.0)	5.7 (4.8-6.8)	0.1 (0.1-0.1)	0.0 (0.0-0.0)	4.5 (3.9-5.1)	0.1 (0.1-0.1)	0.0 (0.0-0.0)	3.4 (3.0-3.7)	0.2 (0.2-0.2)	0.1 (0.1-0.1)	2.5 (2.3-2.7)
CMC+	216,733	0.4 (0.4-0.4)	0.1 (0.1-0.1)	6.7 (5.5-8.0)	0.6 (0.5-0.6)	0.1 (0.1-0.1)	5.1 (4.4-5.8)	0.7 (0.6-0.7)	0.2 (0.1-0.2)	4.2 (3.8-4.8)	0.9 (0.9-1.0)	0.3 (0.3-0.3)	3.1 (2.8-3.4)	1.4 (1.3-1.4)	0.6 (0.5-0.6)	2.5 (2.4-2.7)
IC	43,959	1.7 (1.6-1.8)	0.2 (0.2-0.2)	8.6 (6.9-10.7)	2.4 (2.2-2.5)	0.4 (0.3-0.4)	6.6 (5.6-7.8)	2.9 (2.7-3.0)	0.6 (0.5-0.6)	5.0 (4.4-5.7)	3.9 (3.7-4.0)	1.1 (1.0-1.2)	3.4 (3.1-3.8)	5.2 (5.0-5.4)	2.0 (1.9-2.1)	2.6 (2.4-2.8)
60-64																
CMC-	106,716	0.3 (0.2-0.3)	0.0 (0.0-0.0)	14.4 (9.1-23.0)	0.4 (0.3-0.4)	0.0 (0.0-0.0)	11.1 (7.8-15.6)	0.4 (0.4-0.5)	0.1 (0.0-0.1)	8.2 (6.3-10.9)	0.6 (0.5-0.6)	0.1 (0.1-0.1)	4.8 (3.9-5.8)	0.9 (0.8-0.9)	0.3 (0.2-0.3)	3.2 (2.8-3.7)
CMC+	73,498	1.1 (1.1-1.2)	0.1 (0.1-0.1)	9.8 (7.8-12.2)	1.5 (1.4-1.6)	0.2 (0.2-0.3)	6.2 (5.3-7.2)	1.8 (1.7-1.9)	0.3 (0.3-0.4)	5.1 (4.5-5.9)	2.5 (2.4-2.6)	0.7 (0.7-0.8)	3.4 (3.1-3.7)	3.6 (3.5-3.8)	1.5 (1.4-1.6)	2.5 (2.3-2.6)
IC	16,467	3.3 (3.0-3.6)	0.4 (0.3-0.5)	8.4 (6.5-10.8)	4.6 (4.3-4.9)	0.8 (0.6-0.9)	6.0 (5.0-7.2)	5.4 (5.1-5.8)	1.2 (1.1-1.4)	4.4 (3.8-5.1)	7.2 (6.8-7.6)	2.2 (2.0-2.4)	3.3 (2.9-3.7)	9.7 (9.2-10.1)	3.8 (3.5-4.1)	2.6 (2.3-2.8)
≥65																
CMC-	278,636	1.2 (1.2-1.3)	0.1 (0.1-0.1)	12.2 (10.8-13.7)	1.7 (1.6-1.7)	0.2 (0.2-0.2)	8.4 (7.7-9.2)	1.9 (1.9-2.0)	0.3 (0.3-0.3)	6.4 (6.0-6.9)	2.5 (2.5-2.6)	0.6 (0.6-0.6)	4.2 (4.0-4.4)	3.5 (3.4-3.6)	1.2 (1.2-1.3)	2.8 (2.7-2.9)
CMC+	424,730	4.0 (3.9-4.0)	0.6 (0.6-0.7)	6.1 (5.9-6.4)	5.4 (5.3-5.4)	1.2 (1.2-1.2)	4.5 (4.3-4.6)	6.3 (6.2-6.4)	1.7 (1.7-1.8)	3.6 (3.5-3.7)	8.4 (8.3-8.5)	3.3 (3.2-3.3)	2.6 (2.5-2.6)	11.7 (11.6-11.8)	5.9 (5.8-6.0)	2.0 (2.0-2.0)
IC	89,383	6.2 (6.0-6.3)	1.2 (1.1-1.3)	5.2 (4.9-5.5)	8.6 (8.4-8.8)	2.2 (2.1-2.3)	3.9 (3.7-4.1)	10.2 (10.0-10.4)	3.0 (2.9-3.1)	3.4 (3.2-3.5)	13.4 (13.2-13.7)	5.2 (5.1-5.4)	2.6 (2.5-2.7)	17.9 (17.7-18.2)	8.7 (8.5-8.9)	2.1 (2.0-2.1)
<60																
CMC-	1,179,066	0.1 (0.1-0.1)	0.0 (0.0-0.0)	9.6 (7.5-12.2)	0.1 (0.1-0.1)	0.0 (0.0-0.0)	5.7 (4.8-6.8)	0.1 (0.1-0.1)	0.0 (0.0-0.0)	4.5 (3.9-5.1)	0.1 (0.1-0.1)	0.0 (0.0-0.0)	3.4 (3.0-3.7)	0.2 (0.2-0.2)	0.1 (0.1-0.1)	2.5 (2.3-2.7)
CMC+	216,733	0.4 (0.4-0.4)	0.1 (0.1-0.1)	6.7 (5.5-8.0)	0.6 (0.5-0.6)	0.1 (0.1-0.1)	5.1 (4.4-5.8)	0.7 (0.6-0.7)	0.2 (0.1-0.2)	4.2 (3.8-4.8)	0.9 (0.9-1.0)	0.3 (0.3-0.3)	3.1 (2.8-3.4)	1.4 (1.3-1.4)	0.6 (0.5-0.6)	2.5 (2.4-2.7)
IC	43,959	1.7 (1.6-1.8)	0.2 (0.2-0.2)	8.6 (6.9-10.7)	2.4 (2.2-2.5)	0.4 (0.3-0.4)	6.6 (5.6-7.8)	2.9 (2.7-3.0)	0.6 (0.5-0.6)	5.0 (4.4-5.7)	3.9 (3.7-4.0)	1.1 (1.0-1.2)	3.4 (3.1-3.8)	5.2 (5.0-5.4)	2.0 (1.9-2.1)	2.6 (2.4-2.8)
≥60																
CMC-	385,352	1.0 (0.9-1.0)	0.1 (0.1-0.1)	12.3 (11.0-13.8)	1.3 (1.3-1.3)	0.2 (0.1-0.2)	8.5 (7.8-9.3)	1.5 (1.5-1.6)	0.2 (0.2-0.2)	6.5 (6.1-7.0)	2.0 (2.0-2.0)	0.5 (0.4-0.5)	4.3 (4.0-4.5)	2.8 (2.7-2.8)	1.0 (0.9-1.0)	2.9 (2.8-3.0)
CMC+	498,228	3.6 (3.5-3.6)	0.6 (0.5-0.6)	6.2 (6.0-6.5)	4.8 (4.7-4.9)	1.1 (1.0-1.1)	4.5 (4.4-4.7)	5.6 (5.6-5.7)	1.5 (1.5-1.6)	3.7 (3.6-3.8)	7.6 (7.5-7.6)	2.9 (2.8-2.9)	2.6 (2.6-2.7)	10.5 (10.4-10.6)	5.2 (5.2-5.3)	2.0 (2.0-2.0)
IC	105,850	5.7 (5.6-5.9)	1.1 (1.0-1.1)	5.4 (5.0-5.7)	8.0 (7.8-8.2)	2.0 (1.9-2.1)	4.1 (3.9-4.3)	9.4 (9.3-9.6)	2.7 (2.6-2.8)	3.4 (3.3-3.6)	12.5 (12.3-12.7)	4.7 (4.6-4.9)	2.6 (2.6-2.7)	16.6 (16.4-16.9)	7.9 (7.8-8.1)	2.1 (2.0-2.2)

LRTI: lower respiratory tract infection; RR: relative risk; CMC: chronic medical conditions; IC: immunocompromised

Appendix A. ICD-10 diagnosis codes for LRTI*

Code	Description
J09	Influenza due to certain identified influenza viruses
J09.X1	With pneumonia
J09.X2	With other respiratory manifestations
J09.X3	With gastrointestinal manifestations
J09.X9	With other manifestations
J10	Influenza due to other identified influenza virus
J10.00	With unspecified type of pneumonia
J10.01	With the same other identified influenza virus pneumonia
J10.08	With other unspecified pneumonia
J10.1	With other respiratory manifestations
J10.2	With gastrointestinal manifestations
J10.81	With encephalopathy
J10.82	With myocarditis
J10.83	With otitis media
J10.89	With other manifestations
J11	Influenza due to unidentified influenza virus
J11.00	With unspecified type of pneumonia
J11.08	With specified pneumonia
J11.1	With other respiratory manifestations
J11.2	With gastrointestinal manifestations
J11.81	With encephalopathy
J11.82	With myocarditis
J11.83	With otitis media
J11.89	With other manifestations
J12	Viral pneumonia, not elsewhere classified
J13	Pneumonia due to Streptococcus pneumoniae
J14	Pneumonia due to Haemophiles influenzae
J15	Bacterial pneumonia, not elsewhere classified
J16	Pneumonia due to other infectious organisms, not elsewhere classified
J17	Pneumonia in diseases classified elsewhere
J18 (excl. J18.2)	Pneumonia, unspecified organism (excl. hypostatic pneumonia, unspecified organism)
J20	Acute bronchitis
J21	Acute bronchiolitis
J22	Unspecified acute lower respiratory infection

LRTI: lower respiratory tract infection

*ICD-9 codes available upon request

Appendix B. List of chronic medical conditions and immunocompromising conditions

Comorbidity Profile
CMC+
Cardiovascular (chronic)
Neurologic (chronic)
Hematologic (chronic)
Pulmonary (chronic)
Hepatic (chronic)
Metabolic (chronic)
Renal (chronic)
Cardiopulmonary (chronic)
Obesity
IC
Solid tumor
Hematologic malignances
Solid organ transplant
Other immunosuppressive conditions/treatments

CMC+: persons with chronic medical conditions; IC: persons with immunocompromising conditions and/or treatments

*Persons without any of the above conditions were considered CMC-

Codes are available upon request
